# Newborn Mortality in Sub-Saharan Africa: Why is Perinatal Asphyxia Still a Major Cause?

**DOI:** 10.5334/aogh.2541

**Published:** 2019-08-08

**Authors:** Fatima Usman, Abdulazeez Imam, Zubaida L. Farouk, Aliyu L. Dayyabu

**Affiliations:** 1 Department of Pediatrics, Bayero University Kano, NG; 2 Department of Pediatrics, Aminu Kano Teaching Hospital, Kano, NG; 3 Department of Vaccines and Immunity, Medical Research Council Unit The Gambia at London school of Hygiene and Tropical Medicine, Atlantic Boulevard, Fajara, GM; 4 Department of Obstetrics and Gynecology, Bayero University Kano, NG; 5 Department of Obstetrics and Gynecology, Aminu Kano Teaching Hospital, Kano, NG

## Abstract

Abstract Global new-born mortality has shown steady decline over the last two decades, but this decline has been slowest in Sub-Saharan Africa (SSA). Perinatal asphyxia (PA) is a major cause of new-born deaths in this region and as such SSA now contributes a disproportionate large percentage of global asphyxia-related deaths. In this paper, we examine regional challenges affecting primary, secondary and tertiary prevention of PA and proffers locally adaptable solutions to these identified challenges.

## Background

**Figure 1:**
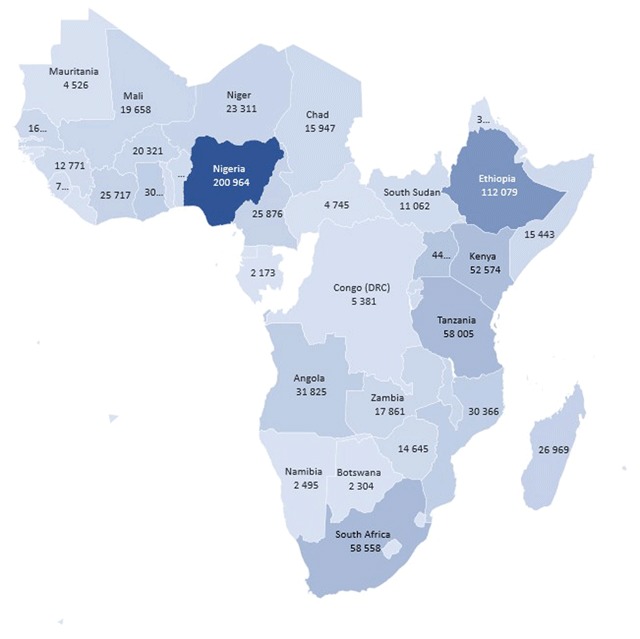
Map showing 46 countries of Sub-Saharan Africa and their populations (in thousands). Data—United Nations World Population Prospects 2019.

**Figure 2:**
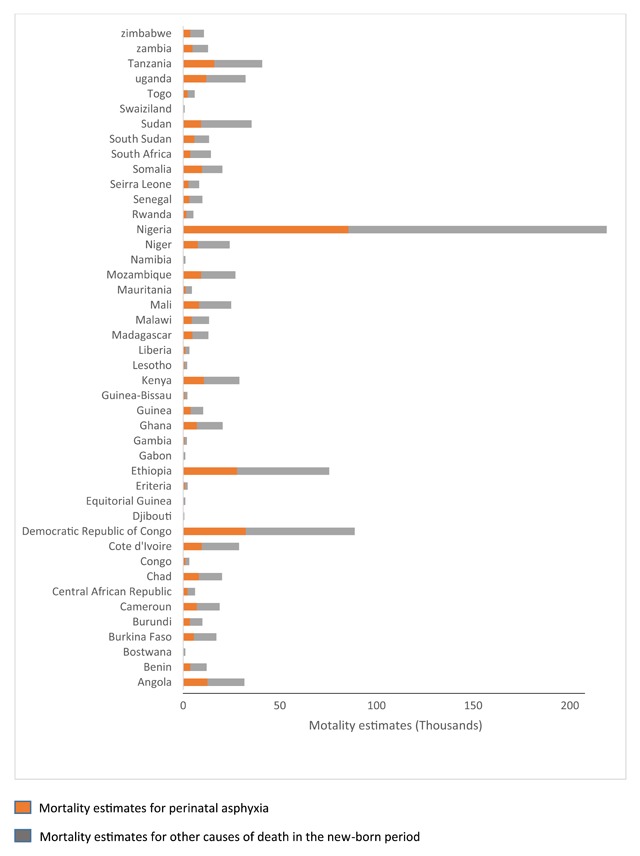
Stacked bar chart showing mortality burden estimates for perinatal asphyxia as a proportion of total neonatal mortality burden for 45 Sub-Saharan African countries. Data Source: Reproduced from World Health Organization Disease burden and mortality estimates: Cause specific mortality 2016.

Global newborn mortality has shown steady decline over the preceding two decades, but this has been slowest in Sub-Saharan Africa (SSA), where newborn deaths now accounts for 38 percent of global neonatal mortality [1]. In this region, the majority of deaths occur largely from preventable causes such as perinatal asphyxia. Asphyxia now accounts for greater than a third of all regional newborn deaths and is associated with significant disabling morbidities among survivors [2]. The region also contributes disproportionately to the global asphyxia mortality burden accounting for 46 percent of these deaths [3]. Countries such has Nigeria and Ethiopia with the greatest population estimates (Figure 1) contribute greatest to the regional burden of asphyxia. Mortality from asphyxia also contributes significantly to neonatal mortality in many countries across the region (Figure 2).

Ending preventable newborn deaths currently feature prominently in the global health agenda and this is reflective in the Sustainable Development Goals, specifically Goal 3 [4]. To achieve these targets however, countries in SSA would need to prevent and better manage complications arising from asphyxia and other common causes of newborn deaths within the region.

In this paper, we examine challenges faced in the prevention and management of perinatal asphyxia by countries within this region, and we suggest possible solutions to the current issues.

## Challenges Affecting Primary Prevention of Perinatal Asphyxia

These challenges relate to identifying and limiting the risk of pregnant women who without intervention might deliver asphyxiated babies. They hinder effective demand and supply of obstetric services within the region.

## Obstetric demand challenges

In SSA, attendance of ante-natal care, which should connect the ‘at-risk pregnant woman’ with the formal health system, is poor, as under 40 percent of women attend at least four visits [5]. Within the region, antenatal coverage varies considerably, and early antenatal visits in the first trimester only occur in a quarter of pregnant women [6]. Countries such as Ghana report coverage rates of over 90 percent, while Mali, Niger, Chad and Ethiopia have around 30 percent [5]. Major contributing factors to this low demand and coverage variability include local health financing models, health facility inaccessibility, socio-cultural norms and low literacy levels [7].

Despite widespread regional poverty, 40 to 54 percent of people access healthcare primarily out-of-pocket which hinders health service demand [8]. In instances where health insurance coverage exists, many families still bear indirect financial burdens for non-existent services which have to be sourced from private facilities [9].

Regional sociocultural norms such as child marriages also negatively affect antenatal care attendance. A recent review of demographic and health survey data in 31 SSA countries documented a third of girls were married by age 18 in more than half of the studied countries [10]. In these societies, healthcare decisions regarding women are made by husbands or prominent male family figures who either do not recognize the importance of such services or are averse to the incurred financial consequences [11]. Other cultural beliefs particular to Western Africa suggest pregnant women are prone to evil spirits, resulting in pregnancy being shrouded in secrecy [12]. In some instances, a local preference for unorthodox obstetric care results in low demand. This is reflective in Ghana, where despite high antenatal coverage, home deliveries are quite common. One study documented almost 90 percent of women who delivered at home had some form of antenatal care visits [13].

## Obstetric supply challenges

These relate to three service delivery gaps: coverage, equity and quality gaps. The coverage gap connotes insufficient manpower to address regional obstetric needs. The global health worker shortage is greatest in SSA, where the majority of countries have health worker to population densities below 2.5 per 1000 population, starkly contrasting developed country estimates which are between two and ten times this number [14]. The inequitable distribution of manpower and other health resources across social class and the rural-urban divide is also a further threat to effective obstetric supply. Skilled birth attendance rates for the regions poorest stand at just a-fifth of the affluent, while only 29 percent of rural deliveries are attended to by skilled workers in contrast to 75 percent in urban areas [15,16].

Additionally, existing quality gaps affect obstetric supply. In a review of data from 20 SSA countries, 71 percent of women did not have intermittent preventive treatment for malaria in pregnancy despite attending at least 4 antenatal care visits [17]. Similarly, 16 and 24 percent respectively did not have their blood and urine samples taken for investigations [17]. An important contributing factor to this quality gap is poor healthcare spending. SSA currently has only one percent of the global financial resources to tackle health related issues [18]. In addition, poor obstetric service organization results in overstretched tertiary health facilities. Such facilities which were ideally built to offer specialized obstetric care, end up providing care across all levels with resultant drop in the quality of care they provide.

## Challenges affecting secondary prevention of perinatal asphyxia

Secondary prevention of asphyxia involves resuscitation of newborn infants with breathing problems who are at risk of developing perinatal asphyxia. One major challenge to this is the large proportion of out-of-hospital births occurring in the presence of unskilled birth attendants who lack knowledge and skill of neonatal resuscitation [19]. More than 60 percent of women deliver at home in urban areas and this figure is considerably higher in the rural parts of SSA [20]. Referral systems connecting local communities and health facilities are often lacking in these settings and families are left to fend for themselves [8].

When births occur in-facility, obstetric service providers frequently do not have the basic skills or equipment necessary to provide quality neonatal resuscitation [21]. A regional survey documented between 2–12 percent of labor room birth attendants to have newborn resuscitation knowledge, and only 8–22 percent of surveyed facilities had appropriate resuscitation equipment [21]. Due to non-availability of basic investigations, definitive diagnosis of asphyxia in these settings is challenging and based on histories of poor crying at birth and low APGAR scores, which have low predictive accuracy [22].

## Challenges affecting tertiary prevention of perinatal asphyxia

Tertiary prevention of asphyxia involves treating acute complications and mitigating their progression to long-term disabling morbidities. This requires functional multidisciplinary rehabilitative care teams which are lacking in SSA. Countries within the region have rehabilitative worker densities below 0.01 per 10,000 population, contrasting developed countries where these figures are almost a thousand times this number [23]. Additionally, due to non-functional vital statistics systems, country-level data on the burden of asphyxia and related impairments required for health planning is virtually non-existent. Available data are either model estimates for burden of disease or hospital admission rates that are not reflective of community prevalence.

## Solutions to the current problem

Focusing on primary prevention within the region is most likely to have the greatest impact on asphyxia burden and this would entail stimulating a demand for obstetric services while improving supply. Infrastructural and human capacity development are key to improving regional obstetric demand. Road networks, transportation, referral, communication and information systems, all of which have direct effects on service demand would need to be upgraded [24]. Additional improvements would be needed in the areas of female child education, female empowerment, average living standards and promotion of sustainable community health insurance schemes [8,9].

Community engagement is also fundamental to stimulating demand and should entail changing community perceptions regarding orthodox obstetric services through health education, local media and provision of holistic, culturally acceptable and easily accessible obstetric services. In these settings, the buy-in of prominent local community, traditional and religious leaders is crucial to programmatic success.

From a supply perspective, an obvious solution to tackle the existing coverage gaps would be to attract skilled birth attendants through competitive remuneration. However, this is not feasible in the short term, as many SSA countries are currently economically challenged. Task-shifting, a practice which involves delegating roles and responsibilities otherwise carried out by senior health staff to lower and mid-health care workers can serve as a short term measure [25]. Local research has shown this to be feasible and effective in multiple settings. A study in Mozambique showed outcomes of Caesarean sections performed by trained medical officers to be similar to those of specialized obstetricians [26]. Other studies from Tanzania, Malawi and Ethiopia, showed no difference in operative outcomes between medical officers and non-physician clinical officers [27,28]. Program managers would however need to ensure supervision and monitoring of the quality of instruction given to these lower cadre staff to ensure the success of such programs.

Equitable distribution of obstetric human resources across geographical divides needs to be ensured through health worker tax rebates and provision of rural allowances to encourage their retention in these areas. Nigeria and Zambia have instituted rural midwives’ and rural health worker retention schemes which have shown modest successes [29,30]. Rural postings for Ghanaian obstetric resident trainees and student mid-wives are mandatory, creating a constant source of obstetric workforce in these communities [31,32]. Access to community health insurance would also reduce the socioeconomic inequity in access to obstetric care.

Regional governments need to increase budgetary allocations to the health sector to improve quality of obstetric care. Local services would also need to be more effectively organized by developing strong bottom-up referral systems which centralize high-risk pregnancies to tertiary obstetric health centers and those considered low-risk to primary healthcare. In addition, proper supervision and monitoring of existing obstetric facilities using methods such as perinatal audits, obstetric checklists, regular in-house staff training and drills will also improve quality of care [33].

Despite being controversial among policy makers [19], training of Traditional Birth Attendants on neonatal resuscitation has shown positive benefits in Asia [21], and this can potentially be adapted and integrated in SSA. Regular trainings on neonatal resuscitation should also be mandatory for skilled obstetric service providers. Neonatal resuscitation courses such as the Helping Babies Breathe course have been shown to be easily adaptable to low-resource countries and can be scaled-up and incorporated into community health programs and nursing school curriculums, with periodic re-certification for trained health care providers [34].

To improve tertiary prevention of asphyxia, capacity building for rehabilitative service providers is essential. Local health facilities would need to establish collaborative links with international agencies and centers with specialized rehabilitative care that can offer voluntary services and develop local capacity. Vital registration of all births and deaths in SSA needs to be improved for accurate data on local asphyxia burden and for managing regional government development plans.

## Conclusion

Challenges to successfully preventing perinatal asphyxia in SSA might seem herculean. The key to solving these would be the institution of pragmatic locally adapted solutions that take into cognizance existing realities in the region. These would need to target safer pregnancy and delivery outcomes with a focus on accessible quality neonatal resuscitation and human capacity development.

## Author Information

Fatima Usman and Abdulazeez Imam Co-wrote first draft and edited final version.

Zubaida Farouk and Aliyu Labaran Dayyabu edited and provided expert perspective throughout the manuscript writing.
